# Unexpected long-term survival of Stage IV pancreatic cancer patient with synchronic liver metastases after multimodal therapy including upfront surgery

**DOI:** 10.1093/jscr/rjac638

**Published:** 2023-01-10

**Authors:** Aiman Obed, Mahmoud Siyam, Amr Anwar Jarrad, Ody Abdelhadi, Muaweih Ababneh, Hassan Annab, Laszlo Füzesi, Abdalla Bashir, Anwar Jarrad

**Affiliations:** Hepatobiliary and Transplant Surgery, Jordan Hospital, Amman, Jordan; General Surgery, Jordan Hospital, Amman, Jordan; Hepatology, Gastroenterology and Hepatobiliary, Jordan Hospital, Amman, Jordan; General Surgery, Jordan Hospital, Amman, Jordan; Department of Anaesthesia, Abdali Hospital, Amman, Jordan; Department of Pathology, Jordan Hospital, Amman, Jordan; Pathology, Faculty of Medicine, University of Augsburg, Augsburg, Germany; General and Transplant Surgery, Jordan Hospital, Amman, Jordan; Hepatology, Gastroenterology and Hepatobiliary/Transplant Unit, Jordan Hospital, Amman, Jordan

## Abstract

We report the case of a 56-year-old male with pancreatic cancer and 25 liver metastases. The patient underwent a distal pancreatectomy with 11 metastasectomies in the left liver lobe. Histological examination demonstrated a moderately differentiated ductal adenocarcinoma with pT3N0M1, Stage IVb. Three weeks later, we performed transarterial chemoembolization for the right lobe of the liver, and after 6 weeks we started systemic chemotherapy with FOLFIRINOX. After 31 months, computer tomography examination showed increases in size of the remaining lesions at segment VII/VIII of the right lobe. All liver metastases were surgically removed and a new chemotherapy was initiated. Nevertheless, after 40 months the patient developed two brain metastases. One was surgically resected and the smaller lesion was treated by gamma knife. Unfortunately, the patient died 42 months after the first presentation. Conclusively, in very selected patients with synchronic liver metastasis, multimodal treatment including repeated surgery, TACE and chemotherapy may prolong survival.

## INTRODUCTION

Pancreatic ductal adenocarcinoma (PDAC) encompasses > 85% of pancreatic cancers and represents one of the most aggressive malignancies with poor prognosis. In all stages, pancreatic cancer is associated with the lowest survival rates of any major cancer type, with 5-year survival rates of <5% [1, 2]. Approximately 50% of patients are diagnosed in Stage IV due to distant metastases at the time of presentation [3]. The metastatic stage of PDAC is the greatest cause of cancer-related deaths, leaving only 1% of patients alive 5 years after their first presentation [3].

Despite the fact that surgical resection offers the best chance for possible cure, only 20% of patients with PDAC may be considered surgical candidates at their first presentation due to locally advanced tumors or distant metastases, such as liver metastases (LMs; [4]). Constant improvement of surgical performance and perioperative patient care has finally allowed surgeons to conduct pancreatic resection with very low mortality rates [4, 5].

## CASE REPORT

**Figure 1:**
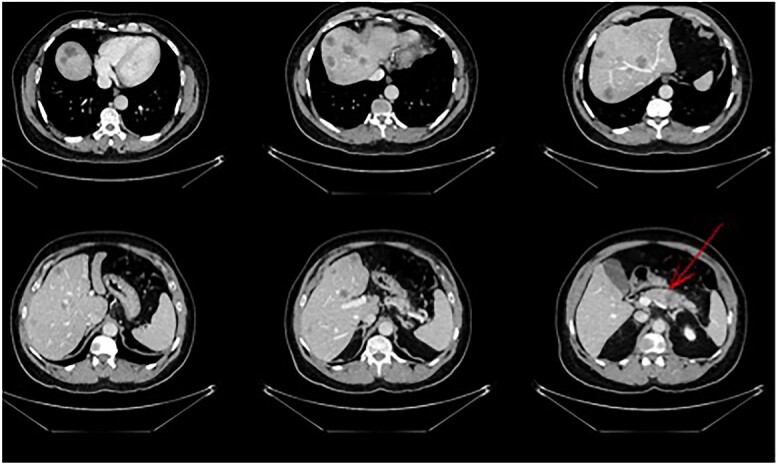
Pancreatic tail/body cancer (red arrow) with multiple liver lesions.

A 56-year-old male in good clinical condition presented at our outpatient clinic with pancreatic-body carcinoma and >25 synchronic LMs. A triple-phase computer tomography (CT) scan of the liver with intravenous contrast confirmed a 2.3-cm ill-defined hypodense lesion in the body of the pancreas and numerous ill-defined lesions, ranging from 1- to 3-cm in size, located in both liver lobes. Small peripancreatic lymph nodes were also noted (Fig. 1). A bone scan and whole-body CT scan were otherwise unremarkable. Serum levels of the tumor markers CA19-9 and CEA were 4730 U/ml and 138 ng/ml, respectively.

The patient was otherwise in good health and willing to undergo treatment beyond guidelines. Our multidisciplinary tumor board suggested a personalized treatment concept with an experimental approach involving four steps.

- Surgery;
- Transarterial chemoembolization of the right lobe;
- Chemotherapy;
- Reassessment amid follow-up.

The patient and his relatives were informed of the new therapeutic and personalized concept. As the first step, we performed an extended distal pancreatectomy and, synchronously, metastasectomy of all 11 lesions in the left lobe. Histologically, moderately differentiated PDAC with pT3N0M1 (HEP) was diagnosed. PD-L1 immunostaining (clone 22c3) revealed a positive score of 60, indicating a low probability of tumor responses with anti-PD-1/L1 antibody treatment. Immunohistochemical expression of DNA mismatch-repair genes was present in the tumor cells, indicating a microsatellite stable tumor.

**Figure 2:**
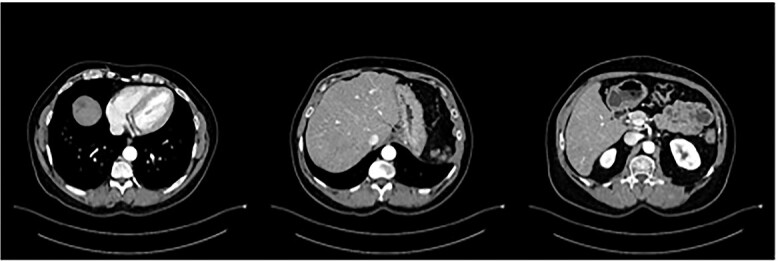
Status post-multimodal therapy with significant lesional downsizing or complete vanishing of cancerous lesions.

**Figure 3:**
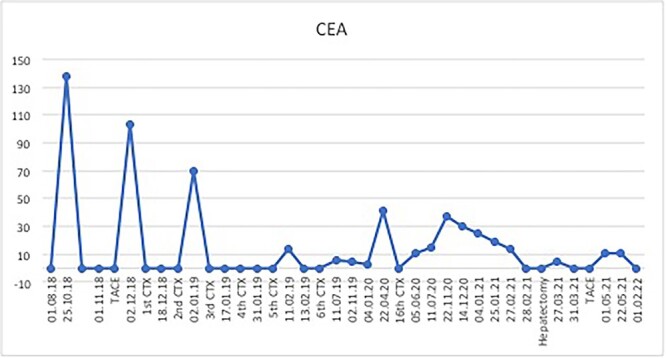
Substantial CEA response after multimodal therapy.

**Figure 4:**
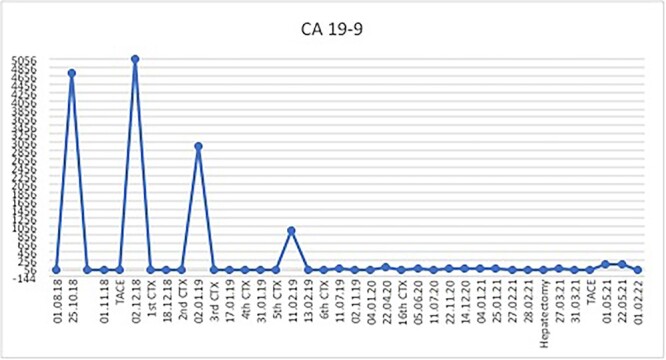
Considerable CA19-9 response after multimodal therapy.

The postoperative course was uneventful and the patient was discharged after 7 days, followed by TACE of the right lobe 3 weeks after surgery. After 6 weeks, the patient received his first dose of the systematic chemotherapy with FOLFIRINOX. A follow-up CT scan confirmed that all remaining LMs in the right lobe had significantly decreased in size or vanished (Fig. 2). Tumor markers (CA19-9 and CEA) decreased continuously to normal levels (Figs. 3 and 4).

Approximately 31 months after resection of the primary tumor and 10 months after ceasing chemotherapy, the patient returned in March of 2021 and was found to have suffered a relapse, suggestive of LM. An abdominal CT scan with intravenous contrast showed multiple lesions in segment VII/VIII. Accordingly, the patient underwent a segmentectomy of segment VII/VIII of the liver. Following the second surgery, additional TACE was performed. A follow-up triple-phase CT scan of the liver showed complete resection of LMs. A small amount of fluid collection around the right lobe was noted and conservatively treated.

**Figure 5:**
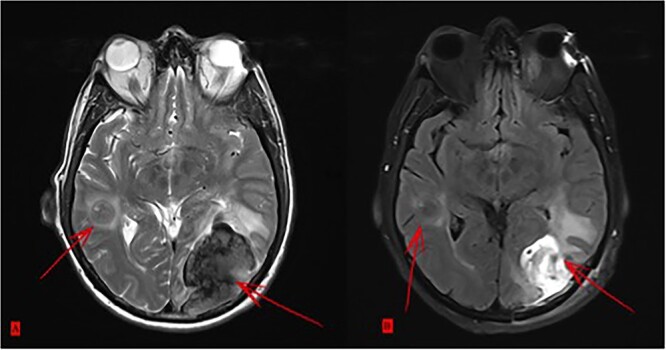
(**A**) Two brain metastases. (**B**) Status after surgical intervention on the bigger lesion.

Postoperatively, tumor marker levels stabilized and a new chemotherapy regime with capecitabine (Xeloda) and gemcitabine (Gemzar) were started. Nonetheless, 40 months after first presenting, the patient developed two brain metastases following an 8-week break from chemotherapy. The larger lesion was surgically resected and the smaller was treated with gamma knife. Histology was consistent with metastatic PDAC (Fig. 5). Unfortunately, the patient died 42 months after first presenting, suffering multiorgan failure. Overall, the patient survived at least 40 months after multimodal therapy with good quality of life, despite the extensive synchronic LMs and the devastating diagnosis.

## DISCUSSION

Many guidelines for pancreatic adenocarcinoma offer suggestions and recommendations for a comprehensive treatment of patients with pancreatic cancer, particularly treatment possibilities for metastatic and locally advanced cancer [6].

Numerous groups have reported enhanced survival for patients who have undergone hepatic resection for non-colorectal and non-neuroendocrine metastases [7, 8].

The most common metastatic organs in PDAC are the liver, peritoneum, lungs and pleura and bones [9]. Increasing support for possible curative liver and lung resection in different tumors is recognized [10], and major surgical resections at primary and metastatic sites have been recognized and adapted for a growing number of tumor types [11]. Nevertheless, reports of surgical resection in pancreatic cancer with synchronous metastases are rare [12]. Currently, guidelines for the treatment of PDAC with LM recommend systematic chemotherapy as the first-line treatment and do not endorse surgery of the primary tumor and synchronous distant metastases without neo-adjuvant treatment. Limited resections of LMs have been widely excluded mainly because PDAC has such a reduced prognosis.

As an additional therapeutic modality in metastatic pancreatic cancer, transarterial chemoembolization (TACE) has been reported in liver metastases from pancreatic adenocarcinoma, mostly in combination with other therapy modalities. TACE may deliver acceptable control of tumor progress and extend survival, especially for patients with multiple liver metastases [13].

Immune checkpoint blockades affecting programmed cell death protein 1 PD-1 ligand 1 (PD-L1) are now the most common armament in tumor immunotherapy. Medical studies have also shown that the administration of an anti-PD-L1 monoclonal antibody results in poorer outcomes in pancreatic tumor cases [14].

Su *et al*. concluded that surgical treatments for primary tumors and metastatic sites enhanced survival, as synchronous resection of pancreatic tumors and hepatic metastases presented a significant survival advantage in well-selected patients [15].

Further studies are needed to determine the best treatment plan for selected patients who can benefit from synchronic resection of primary PDAC and LM. Potential selection criteria may include <60 years of age, good general health (i.e. cardio-pulmonary function) and consent to undergo treatment beyond guidelines.

In conclusion, a comprehensive multimodal treatment including upfront/repeated surgery, TACE and chemotherapy might result in prolonged survival in carefully selected patients.

## DATA AVAILABILITY

Data sharing is not applicable to this article.

## CONSENT FOR PUBLICATION

Written informed consent was obtained from the patient for the publication of this report.

## CONFLICT OF INTEREST STATEMENT

The authors have no conflicts of interest to disclose. All authors have given their final approval of the submitted version.

## FUNDING

None.

## References

[1] Antoniou E , Margonis GA, Sasaki K, Andreatos N, Polychronidis G, Pawlik TM, et al. Is resection of pancreatic adenocarcinoma with synchronous hepatic metastasis justified? A review of current literature. ANZ J Surg 2016;86:973–7. 27580713 10.1111/ans.13738

[2] Winter JM , Cameron JL, Campbell KA, Arnold MA, Chang DC, Coleman J, et al. 1423 pancreaticoduodenectomies for pancreatic cancer: a single-institution experience. J Gastrointest Surg 2006;10:1199–211. 17114007 10.1016/j.gassur.2006.08.018

[3] Pawlik TM , Gleisner AL, Cameron JL, Winter JM, Assumpcao L, Lillemoe KD, et al. Prognostic relevance of lymph node ratio following pancreaticoduodenectomy for pancreatic cancer. Surgery 2007;141:610–8. 17462460 10.1016/j.surg.2006.12.013

[4] Takada T , Yasuda H, Amano H, Yoshida M, Uchida T. Simultaneous hepatic resection with pancreato-duodenectomy for metastatic pancreatic head carcinoma: does it improve survival? Hepatogastroenterology 1997;44:567–73. 9164539

[5] Karandish F , Mallik S. Biomarkers and targeted therapy in pancreatic cancer. Biomark Cancer 2016;8:27–35. 27147897 10.4137/BiC.s34414 PMC4847554

[6] Tempero MA , Malafa MP, Al-Hawary M, Behrman SW, Benson AB, Cardin DB, et al. Pancreatic adenocarcinoma, version 2.2021, NCCN clinical practice guidelines in oncology. J Natl Compr Canc Netw 2021;19:439–57. 33845462 10.6004/jnccn.2021.0017

[7] Laurent C , Rullier E, Feyler A, Masson B, Saric J. Resection of noncolorectal and nonneuroendocrine liver metastases: late metastases are the only chance of cure. World J Surg 2001;25:1532–6. 11775186 10.1007/s00268-001-0164-7

[8] Yedibela S , Gohl J, Graz V, Pfaffenberger MK, Merkel S, Hohenberger W, et al. Changes in indication and results after resection of hepatic metastases from noncolorectal primary tumors: a single-institutional review. Ann Surg Oncol 2005;12:778–85. 16132374 10.1245/ASO.2005.11.018

[9] Embuscado EE , Laheru D, Ricci F, Yun KJ, de Boom WS, Seigel A, et al. Immortalizing the complexity of cancer metastasis: genetic features of lethal metastatic pancreatic cancer obtained from rapid autopsy. Cancer Biol Ther 2005;4:548–54. 15846069 10.4161/cbt.4.5.1663 PMC2771924

[10] Cheung FP , Alam NZ, Wright GM. The past, present and future of pulmonary metastasectomy: a review article. Ann Thorac Cardiovasc Surg 2019;25:129–41. 30971647 10.5761/atcs.ra.18-00229 PMC6587129

[11] Silberhumer GR , Paty PB, Denton B, Guillem J, Gonen M, Araujo RLC, et al. Long-term oncologic outcomes for simultaneous resection of synchronous metastatic liver and primary colorectal cancer. Surgery 2016;160:67–73. 27079362 10.1016/j.surg.2016.02.029 PMC4911703

[12] Gleisner AL , Assumpcao L, Cameron JL, Wolfgang CL, Choti MA, Herman JM, et al. Is resection of periampullary or pancreatic adenocarcinoma with synchronous hepatic metastasis justified? Cancer 2007;110:2484–92. 17941009 10.1002/cncr.23074

[13] Kotoyan R , Metzger T, Tatum C, Robbins K, Martin RC 2nd. Hepatic arterial therapy with drug-eluting beads in the management of metastatic pancreatic carcinoma to the liver: a multi-institutional registry. J Oncol 2012;2012:168303. 22481917 10.1155/2012/168303 PMC3306937

[14] Winograd R , Byrne KT, Evans RA, Odorizzi PM, Meyer AR, Bajor DL, et al. Induction of T-cell immunity overcomes complete resistance to PD-1 and CTLA-4 blockade and improves survival in pancreatic carcinoma. Cancer Immunol Res 2015;3:399–411. 25678581 10.1158/2326-6066.CIR-14-0215 PMC4390506

[15] Yang J , Zhang J, Lui W, Huo Y, Fu X, Yang M, et al. Patients with hepatic oligometastatic pancreatic body/tail ductal adenocarcinoma may benefit from synchronous resection. HPB (Oxford) 2020;22:91–101. 31262486 10.1016/j.hpb.2019.05.015

